# Responses of leaf anatomy, growth, and physiology in backcross 1 progeny from* Saccharum* spp. hybrids and *S. spontaneum* under drought stress at early growth phase

**DOI:** 10.7717/peerj.20822

**Published:** 2026-02-19

**Authors:** Kanlayanee Wiangwiset, Patcharin Songsri, Nakorn Jongrungklang

**Affiliations:** 1 Department of Agronomy, Faculty of Agriculture, Khon Kaen University, Khon Kaen, Thailand; 2 Northeast Thailand Cane and Sugar Research Center, Faculty of Agriculture, Khon Kaen University, Khon Kaen, Thailand

**Keywords:** Stomatal crypt depth, Bulliform cells, Transpiration, Drought stress, Sugarcane

## Abstract

A deeper understanding of the anatomical, physiological, and morphological responses of interspecific hybrid sugarcane to water deficit conditions could enhance the efficiency of developing drought-resistance varieties. This study aimed to investigate the responses of anatomical characteristics of backcross (BC) 1 interspecific hybrids, produced by crossing commercial cane with wild-type sugarcane, under drought and well-watered treatment. A two—factorial pot experiment in a completely randomized design (CRD) with three replications was conducted. Soil volume water contents were 12.34% prior to the start of drought treatment. Two water regimes, field capacity (FC) and drought stress (DS), were tested, with the DS treatment involving water withholding for 51 st to 110 th days after planting (DAP). Fifteen sugarcane genotypes were evaluated, including ten BC1 interspecific hybrids, two commercial varieties (F152 and UT5), the wild type (ThS98-94), the F1 interspecific hybrid male parent (F4-19), and KK3 as a drought resistance check. Anatomical, physiological, and morphological traits were measured at 110 DAP; results indicated that BC1-1-7, BC1-1-44, and BC1-1-50 exhibited superior traits than the other BC1 interspecific hybrids in this study. These genotypes had a high morphological drought resistance index (DRI) for tiller height and tiller number, and the anatomical leaf had low leaf thickness (% reduction from FC by 14.0%, 10.0% and 3.9%, respectively) and vertical length of bulliform cells (% reduction from FC by 26.2%, 23.9% and 4.9%, respectively) but high cuticle thickness (an increase from FC by 10.7%, 18.3% and 19.7%, respectively) and stomatal crypt depth (an increase from FC by 21.0%, 12.9% and 13.4%, respectively), resembling those of the wild type and F1 interspecific hybrid. Additionally, BC1-1-7 demonstrated greater drought resistance, characterized by low stomatal conductance (% reduction from FC by 90.6%), reduced transpiration rates (% reduction from FC by 89.9%), and high water use efficiency (% gain from FC by 76.1%) compared to BC1-1-44 and BC1-1-50. These results highlight the importance of genetic diversity in breeding programs, as interspecific hybrids like BC1-1-7 combine traits from wild and cultivated relatives, enhancing drought stress performance. Future research should evaluate BC1 hybrids under drought stress in the field, focusing on agronomic performance and final yields.

## Introduction

Sugarcane (*Saccharum* spp.), a leading global sugar crop, contributes approximately 80% of the world’s sugar production (Chen et al., 2021) and is pivotal in bioethanol production, with nearly 50% of the crop utilized for ethanol (Joglekar et al., 2022). Sugarcane produces valuable by-products like molasses and bagasse, driving economic benefits across the supply chain (Formann et al., 2020). Sugarcane growth and development are intrinsically linked to yield components and physiological traits (Nawae et al., 2020). Environmental factors such as temperature, light, humidity, and soil quality significantly impact plant growth (Heath et al., 2020). Drought stress, a major environmental challenge, adversely affects plant development, often causing temporary or permanent wilting (Mall et al., 2020; Gomathi et al., 2020). In tropical regions, sugarcane cultivation primarily relies on rain-fed conditions. The variability in the amount and distribution of rainfall leads to drought stress, which significantly impact sugarcane yield (Khonghintaisong, Songsri & Jongrungklang, 2021). Sugarcane faces significant productivity challenges, with a water deficit causing up to a 60% reduction in crop yield (Gomathi et al., 2020). Critical growth phases, particularly between 60–150 days after planting (DAP), are adversely affected by drought in terms of tillering (Inman-Bamber & Smith, 2005). Drought-induced adaptations disrupt essential processes such as photosynthesis and transpiration (Yang et al., 2021), complicating the study and characterization of drought tolerance, a trait regulated by numerous genes (Zia et al., 2021). These strategies involve reducing leaf area, inducing leaf rolling, decreasing stomatal size and density, shrinking epidermal cells, making cell walls more flexible, strengthening the cuticle, and increasing lignin production (Nasir, Shahzadi & Nawaz, 2023). These adaptations help plants stay metabolically stable and cope with drought stress (Mishra & Pandey, 2022).

Sugarcane productivity is significantly constrained by drought stress, particularly in regions with limited water availability (Dlamini, 2021). While commercial cultivars exhibit high yield potential, they often lack the requisite drought resistance, resulting in substantial yield losses under water-deficit conditions (Mahadevaiah et al., 2021). Interspecific hybridization, which integrates the high-yield potential of commercial *Saccharum* hybrids with the intrinsic drought tolerance of wild relatives such as *S. spontaneum*, presents a promising strategy for enhancing drought resilience in elite varieties (Alwala et al., 2009). However, despite its potential, the specific anatomical, physiological, and growth responses of backcross 1 (BC1) progeny under drought stress remain largely unexplored. Investigating these responses during early developmental stages is crucial, as the establishment of efficient physiological adaptations at this phase plays a pivotal role in determining overall plant resilience and yield potential (Reyes et al., 2021).

In *Saccharum*, *S. spontaneum* exhibits greater genetic diversity compared to the predominantly cultivated *S. officinarum*, particularly in traits related to abiotic stress resistance and genomic composition (Xiong, Chen & Shi, 2022). Currently, sugarcane breeding and cultivar development focus on enhancing drought tolerance and high–yield potential. This involves generating inter-specific hybrids by crossing commercial sugarcane varieties with wild relatives, such as *S. spontaneum.* The goal is to develop cultivars that are drought-tolerant, disease and pest-resistant, fast-growing, with robust, deep root systems, and high yields (Perera et al., 2022). However, sugarcane breeding programs face challenges in enhancing genetic diversity and developing new varieties due to the highly heterozygous and complex nature of the sugarcane genome. The polyploid nature of modern sugarcane cultivars, derived from interspecific hybridization, further complicates the breeding process. Crossing *S. officinarum* and *S. spontaneum* produces F1 hybrid sugarcane with high yield and increased drought resistance but low sweetness, high tiller numbers, and low weight per stalk, resulting in low overall yield (Jumkudling et al., 2022). Commercial sugarcane varieties, however, have high sweetness, fewer tillers, and higher weight per stalk, leading to higher yields. To address these issues, backcrossing (BC) is performed, where the F1 hybrid is backcrossed with cultivated varieties to enhance sweetness traits and reduce unfavorable wild-type characteristics (Reyes-Valdés, 2000; Yu et al., 2022). This approach not only improves yield and sweetness but also expands genetic diversity in sugarcane breeding.

Previous research has explored various aspects of the agronomic, anatomical, and physiological responses of sugarcane. Drought stress adversely affects photosynthesis in sugarcane, reducing both quality and yield (Yang et al., 2022). Drought-tolerant sugarcane varieties exhibit traits such as increased cuticle thickness, vascular bundle development, stomatal density, leaf thickness, and epidermal tissue cells, as well as reduced leaf area and smaller stomatal size (Taratima et al., 2019; Taratima et al., 2020; Khonghintaisong, Songsri & Jongrungklang, 2023). These characteristics help reduce photosynthesis and transpiration rates, thereby acclimating to drought stress. Another adaptive mechanism to reduce water loss under soil moisture constraints involves increasing the resistance to water flow from the stomata. Furthermore, a greater stomatal crypt depth helps minimize water loss (Jumkudling et al., 2022). In addition, physiological responses of commercial sugarcane varieties to moisture deficit stress indicate that relative water content and chlorophyll content are effective indicators of stress tolerance (Dapanage & Bhat, 2017). A study by Jumkudling et al. (2022) examined leaf thickness, cuticle thickness, vertical length of bulliform cells, and stomatal crypt depth under supplemented watering conditions, revealing substantial diversity and distribution of these traits among F1 hybrid clones. Backcrossing F1 interspecific hybrids with commercial canes resulted in slightly higher averages for leaf anatomical traits related to drought tolerance, such as stomatal crypt depth, bulliform cell percentage, and cuticle thickness (Wiangwiset et al., 2023a; Wiangwiset et al., 2023b). This indicates successful introgression of drought-tolerant anatomical properties from wild species into F1 hybrids and subsequently into BC1 populations. These anatomical changes are indicative of the drought stress response and can serve as important markers for assessing drought tolerance in sugarcane.

A better understanding of the anatomical, physiological, and morphological responses of interspecific hybrid sugarcane to drought could facilitate the development of drought-resistant varieties. The physiological impacts of drought on various sugarcane genotypes, including hybrid and backcross lines, have been well-documented, highlighting differences in drought tolerance, particularly regarding photosynthetic activity and biomass yield (Tippayawat et al., 2023). However, there is a lack of reports on the responses of leaf anatomy to drought stress. However, the anatomical traits related to drought resistance in backcross hybrids have not been extensively studied. Previous research has only examined the genetic diversity and population distribution of F1 hybrids resulting from crosses between *Saccharum* hybrid spp. and *S. spontaneum*. This research aims to elucidate the responses of BC1 hybrids to drought stress, focusing on anatomical, physiological, and growth traits. By elucidating the intricate interactions between leaf anatomy, growth dynamics, and physiological responses in BC1 progeny derived from interspecific hybridization between *Saccharum* spp. hybrids and *S. spontaneum* under early-stage drought stress, this study enhances our understanding of drought resistance mechanisms. These findings provide a crucial foundation for the development of sugarcane cultivars optimized for sustainable production in water-limited environments.

## Materials & Methods

### Planting materials

This study utilized fifteen sugarcane genotypes, including ten BC1 interspecific hybrid clones (BC1-1-7, BC1-1-21, BC1-1-36, BC1-1-42, BC1-1-44, BC1-1-50, BC1-1-63, BC1-1-65, BC1-1-68, and BC1-1-81), two commercial cane varieties (F152 and UT5), ThS98-94 as the wild type, F1 interspecific hybrid F4-19 as the male parent (had low leaf thickness (LT) but high stomatal crypt depth (SCD) and cuticle thickness (CT)) (Wiangwiset et al., 2023a; Wiangwiset et al., 2023b), and KK3 as a commercial drought-resistant check. The BC1 hybrids were developed by the Department of Agriculture (DOA) Thailand, using an F1 interspecific hybrid derived from *Saccharum* spp. hybrid (F152) and *S. spontaneum* (ThS98-94).

BC1 interspecific hybrids were developed by crossing an interspecific F1 clone with commercial sugarcane. The F1 interspecific hybrid (F4-19), used as the male parent, exhibited high fiber content, tiller number, and stalks per clump but had low sweetness and small stalk diameter. It was crossed with the commercial cane genotype UT5, chosen for its high sugar content and yield.

### Site description and experimental design

This study was conducted at the Agronomy Research Station (16°28′N, 102°48′E, 200 m above sea level) at Khon Kaen University, Thailand, from 27 November 2022 to 16 March 2023, during the dry season in Thailand. The open field pot experiment used a 2  × 15 factorial combination of water regimes and sugarcane genotypes in a CRD with three replications. Factor A consisted of two water regimes: field capacity (FC) and drought stress (DS). Factor B included fifteen diverse sugarcane genotypes.

### Management practices

A set of sugarcane was planted and germinated in plastic bags, and 20 days after planting (DAP), healthy, uniform seedlings were selected and transplanted into large cement pots (75 cm in diameter, 100 cm in height) filled with 556.4 kg of Yasothon series soil (fine-loamy; siliceous, isohypothermic, Oxic Paleustults; bulk density of 1.4 g cm^−^^3^), up to a height of 90 cm. Before transplanting, a mixed chemical fertilizer (47 kg N, 47 kg P_2_O_5_, 47 kg K_2_O per hectare) was applied. During early development, weeds were controlled by hand weeding, and pests and diseases were managed only if they exceeded the economic injury level (EIL) (White et al., 2008).

Water was usually applied to maintain the soil moisture 12.34% (field capacity (FC)) value at FC level throughout experiment for FC treatment, and the soil moisture content was controlled with not more or less than 1% from FC level. Crop water requirement of sugarcane was calculated following (Jangpromma et al., 2012) formula (Jangpromma et al., 2012):

\begin{eqnarray*}\text{ET crop}={\mathrm{ET}}_{\mathrm{o}}\times {\mathrm{}K}_{\mathrm{c}} \end{eqnarray*}

where crop evapotranspiration (ET crop_)_ is the crop water requirement (mm day^−1^), ET_o_ is the evapotranspiration of a reference crop under specified condition calculated by evaporation pan method (ET_o_ = K_p_ × Epan; K_p_ = pan coefficient (class A plan with green fetch) and Epan=pan evaporation (mm day^−1^), K_c_ isthe crop water requirement coefficient for sugarcane (Khonghintaisong, Songsri & Jongrungklang, 2021).

For the DS treatment, water was applied based on crop water requirements from transplanting to 51 days after planting (DAP) to ensure seedling uniformity. From 51 to 110 DAP, water was withheld to mimic drought stress during the early growth stage.

### Meteorological conditions and soil moisture content

**Figure 1:**
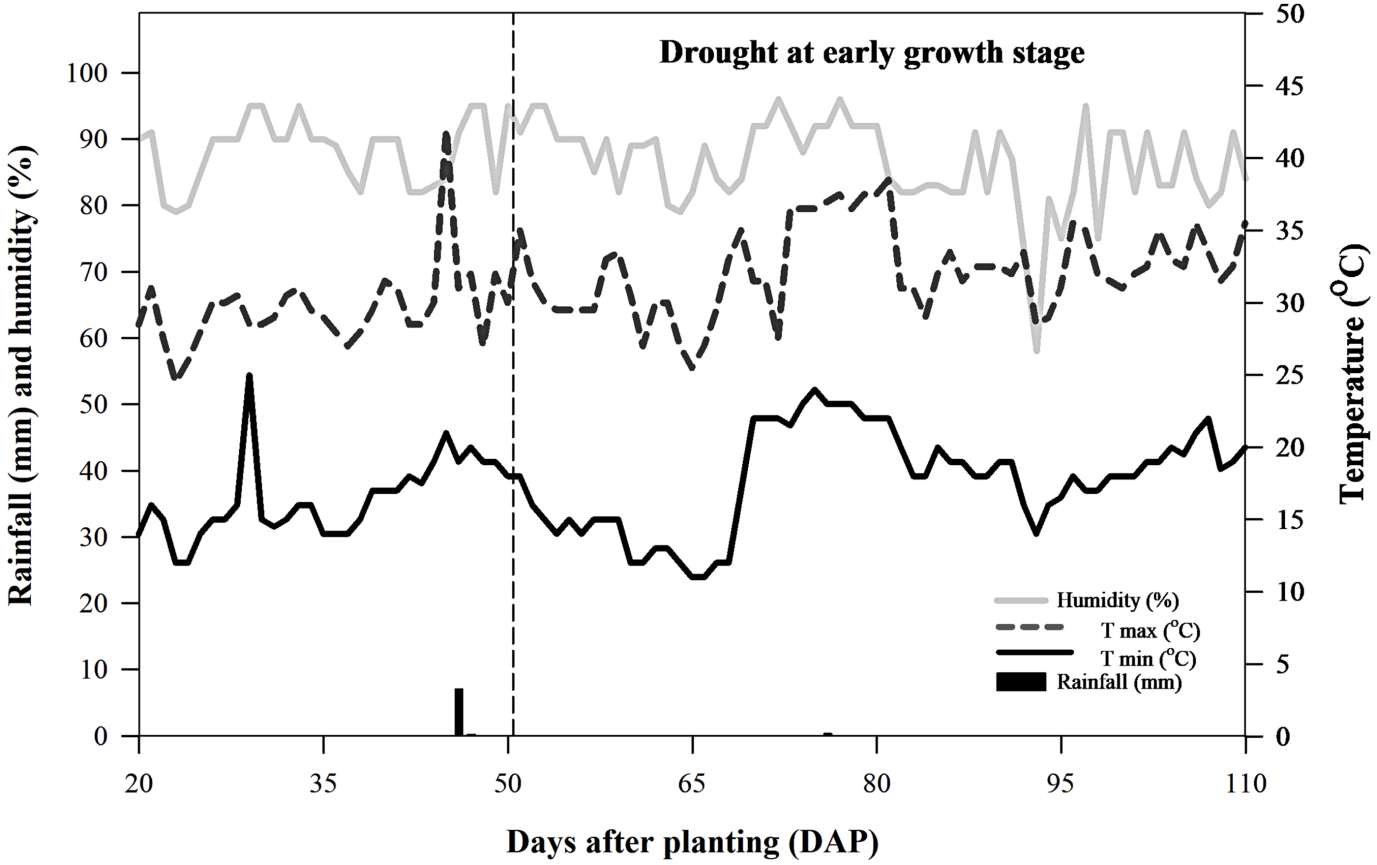
Rainfall (mm), relative humidity (%), maximum daily temperature (°C), and minimum daily temperature (°C) throughout the experimental period for sugarcane.

Daily records of rainfall, relative humidity, and maximum and minimum temperatures were taken from 27 November 2022 to 16 March 2023 at the Agronomy Research Station, Faculty of Agriculture, Khon Kaen University, approximately 1.5 km from the experimental site. The total rainfall during the experiment from 16 December 2022 to 16 March 2023 was 7.5 mm, with only 0.3 mm falling during the drought stimulation period. Therefore, the rainfall in this experiment did not affect growth during the drought stress period. Daily relative humidity ranged from 58.0% to 96.0%, while minimum and maximum daily temperatures ranged from 11.0 °C to 26.0 °C and 24.5 °C to 42.0 °C, respectively (Fig. 1). During the drought stress period from January to February 2023, daily relative humidity remained between 58.0% and 96.0%, with minimum and maximum daily temperatures ranging from 11.0 °C to 24.0 °C and 25.5 °C to 38.5 °C, respectively (Fig. 1). The soil used in this experiment was classified as Yasothon series, characterized as loamy sand, with 77.93% sand, 20.0% silt, and 2.07% clay. Its chemical properties included a pH of 6.46, cation exchange capacity of 13.48 cmol kg^−1^, 0.38% organic matter, 0.358% total nitrogen, 0.180 mg/kg available phosphorus, 32.08 mg/kg exchangeable potassium, and 12.34% field capacity.

Soil moisture content was measured at 51, 67, 81, 95, and 110 DAP using the gravimetric method at depths of 0–30 cm and 30–60 cm. Soil samples were weighed and oven-dried at 105 °C for 72 h, and the percentage of soil moisture was calculated from the wet and dry soil weights using the following formula):

\begin{eqnarray*}\text{soil moisture content}~(\%)= \left[ \frac{\text{wet soil}-\text{dry soil}}{\text{dry soil}} \right] \times 100. \end{eqnarray*}

**Figure 2:**
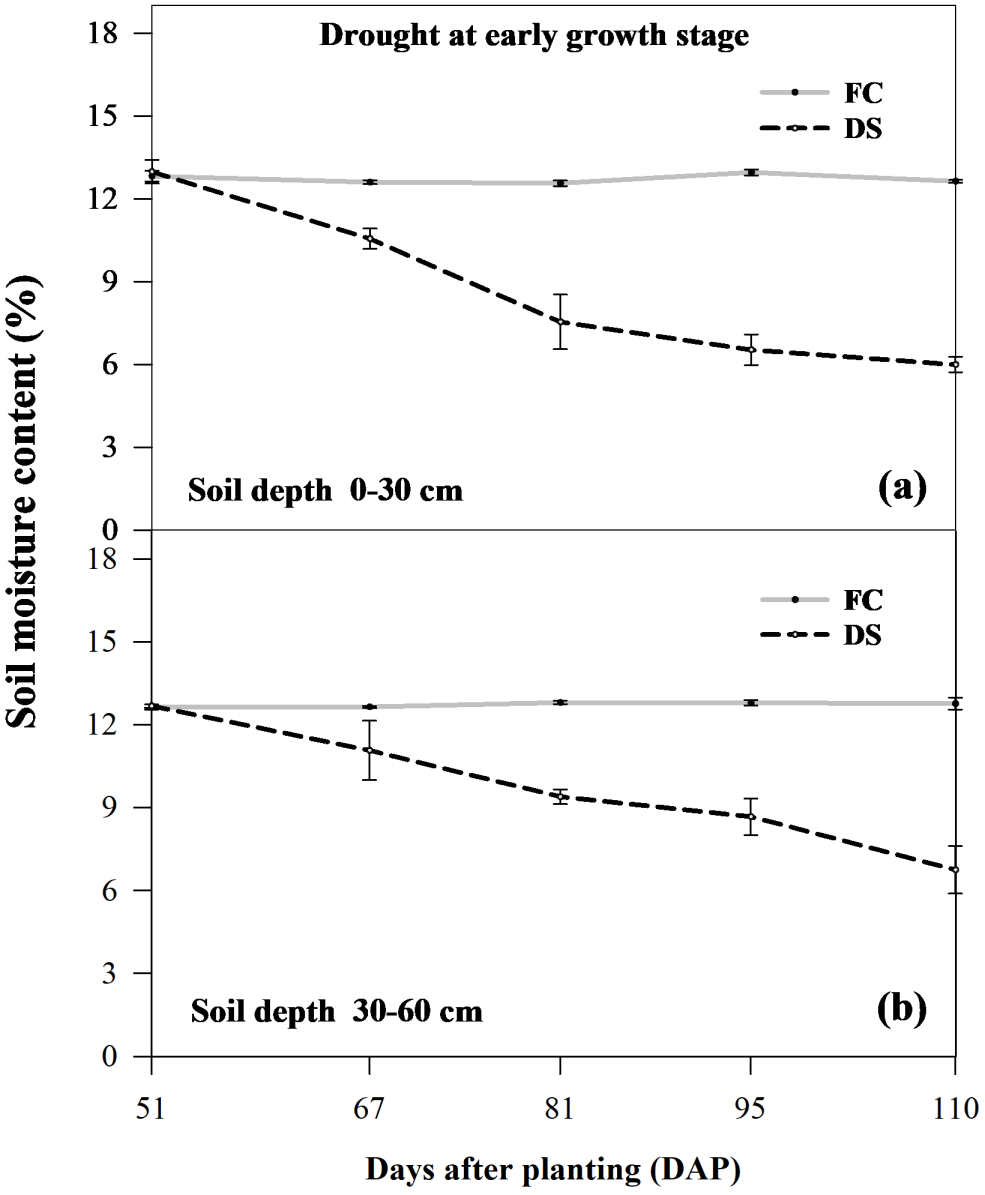
Soil moisture content (%) at depths of 0–30 cm (A) and 30–60 cm (B) below the soil surface under FC and DS conditions in sugarcane during 51 to 110 days after planting (DAP). FC, field capacity and DS, drought stress.

Two water regimes, FC and DS treatments, were applied to all sugarcane cultivars, significantly altering soil moisture content. The soil moisture content determined from soil physical properties was 12.34% for field capacity and 5.75% for the permanent wilting point. Throughout the experiment, the soil moisture content in the FC treatment ranged from 12.56% to 12.95%. During the 81 DAP to 110 DAP in DS treatments, the soil moisture content at depths of 0–30 cm and 30–60 cm gradually dropped from 7.54% to 6.00% and from 9.39% to 6.75%, respectively (Fig. 2). The moisture content of the soil receiving additional irrigation water (under FC conditions) remained close to FC. Soil moisture content measurements confirmed the adequate control of irrigation applications (Fig. 2).

### Data collections

#### Growth traits

After transplanting, plant height (measured from the soil surface to the dewlap of the main tiller) and tiller number were recorded for each pot. These non-destructive measurements were taken at 110 DAP.

#### Physiological traits

##### Photosynthesis.

Leaf gas exchange parameters were measured on the third fully expanded leaf from the top of the main stem at 110 DAP. The physiological parameters, including stomatal conductance (*g*s, mol m^−2^s^−1^), transpiration rate (*E*, mmol H_2_O m^−2^s^−1^) and net photosynthetic rate (*P*n, µmol m^−2^s^−1^) were recorded. Measurements were taken in three replicates, with one plant per replicate per treatment. The net photosynthetic rate and gas exchange parameters were measured and automatically calculated using a LI-COR 6800 Portable Photosynthesis System (LI-6800; LI-COR Biosciences, Lincoln, NE, USA). The photosynthetic parameters were analyzed between 11:00 to 14:00 am. The following conditions were used: 400 µmol mol^−1^ CO_2_ concentration, 1,500 µmol m^−2^s^−1^ light intensity, 500 µmol s^−1^ flow rate, and an air temperature of 30 °C. The sample humidity was controlled to ensure it did not exceed 40%.

##### Water use efficiency (WUE).

The intrinsic water use efficiency (µmol mmol^−1^) was estimated using calculated from the derived transpiration rate (*E*), and net photosynthetic rate (*P*n). The formula was based on Silva et al. (2013) as follows:

\begin{eqnarray*}\text{water use efficiency}~ \left( \mathrm{WUE} \right) = \frac{\text{Net photosynthetic rate}~(P\mathrm{n})}{\text{Transpiration rate}~(E)} . \end{eqnarray*}

##### SPAD chlorophyll meter reading (SCMR).

Leaf greenness was non-destructively measured on the third fully expanded leaf from the top of the main stem using a SPAD-502 chlorophyll meter (Minolta SPAD-502; Minolta, Tokyo, Japan) between 09:00 and 12:00 at 110 DAP.

##### Relative water content (RWC).

Five leaf discs per plant at 110 DAP were collected, immediately sealed in glass vials, and quickly transported to the laboratory in an ice-cooled chest. Leaf disc fresh mass were determined within 2 h of excision. The turgid weight was obtained after rehydration in deionized water for 24 h at room temperature. After rehydration, the discs were quickly and carefully blotted dry with lint-free tissue paper before determining the turgid weight. The dry weights were recorded after drying in the heat drier at 80 °C for 48 h (Silva et al., 2014).

\begin{eqnarray*}\mathrm{RWC}~(\%)= \frac{(\text{Leaf fresh weight}-\text{Leaf dry weight})}{(\mathrm{Leaf}~\mathrm{turgid}~\mathrm{weight}-\text{Leaf dry weight})} \times 100. \end{eqnarray*}

#### Anatomical traits

**Figure 3:**
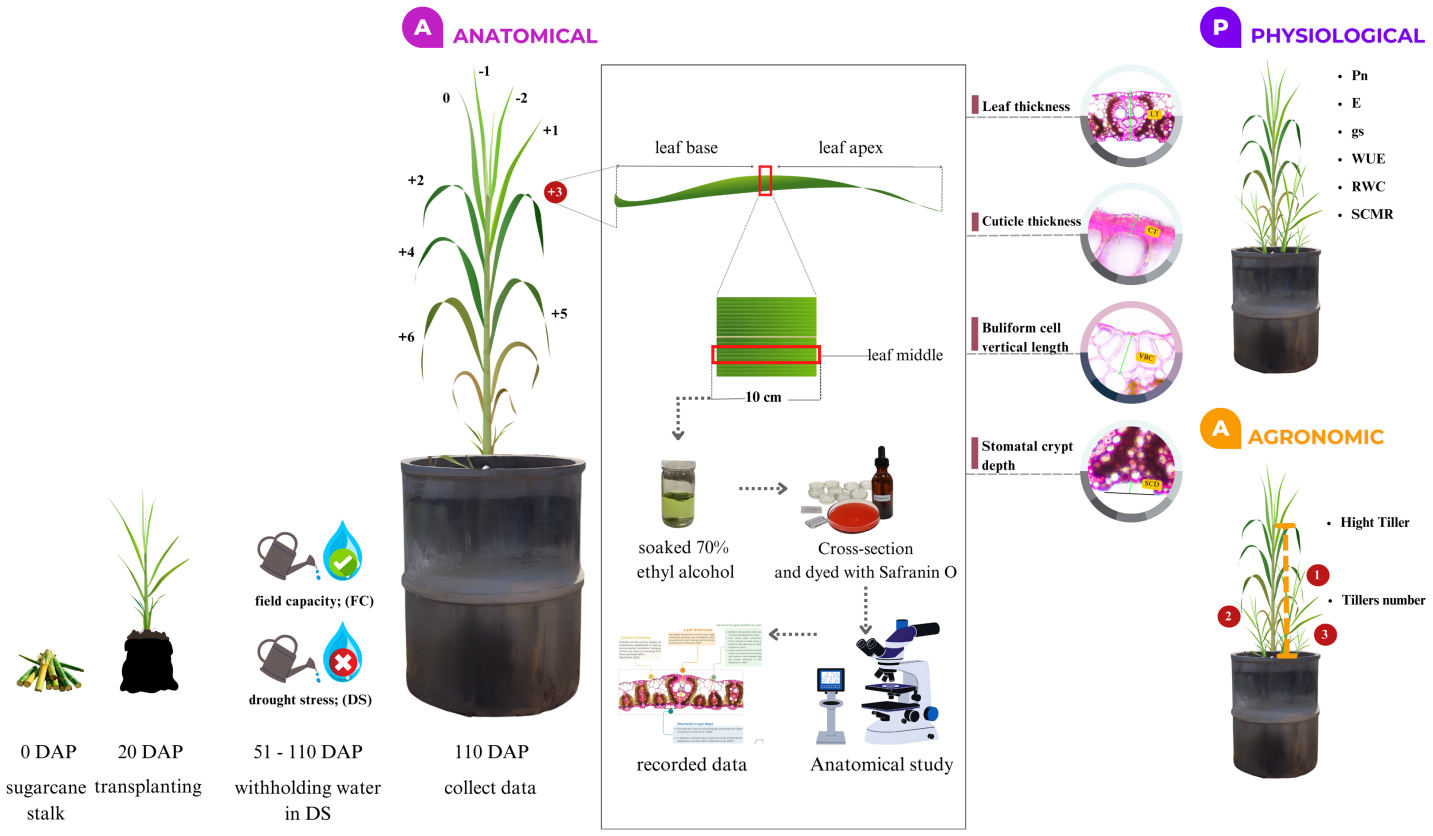
The study characterized the anatomical features of mature sugarcane leaves, focusing on selected cuts. Parameters assessed included LT, CT, VBC, and SCD LT, leaf thickness; CT, cuticle thickness; VBC, vertical length of bulliform cells; and SCD, stomatal crypt depth.

The third leaf of the main tiller was collected at 110 DAP and sampled with three repetitions. Leaf length was measured, and each leaf sample was cut into a 10 cm segment from the middle part (Fig. 3). The samples were immediately soaked in 100 ml of 70% ethyl alcohol for 48 h to preserve and stabilize the cells for anatomical studies. Leaf sections were dissected into small pieces and sectioned by hand to create the thinnest possible slices. The tissue was placed on a slide, stained with 1% (w/v) Safranin O, dehydrated with a series of ethyl alcohol and xylene washes, and mounted using DePeX mounting medium (for mounting plant specimens for anatomical and histological analysis). The slides were then mounted with distilled water for further anatomical studies.

In this study, leaf anatomy was quantitatively characterized, focusing on traits such as leaf thickness (LT), cuticle thickness (CT), vertical length of bulliform cells (VBC), and stomatal crypt depth (SCD) (Fig. 3). Anatomical features were studied and recorded using an Olympus BH-2 light compound microscope (Olympus Corporation, Tokyo, Japan) and a Zeiss 540214-0000004 with the MB2004 configuration Axio Vision (MB2004 configuration-AV) program (Carl Zeiss Microscopy GmbH, Jena, Germany) at 10X magnification.

#### Drought resistance index

The DRI was calculated to compare the values between drought stress (DS) and field capacity (FC) treatments. The formula was described by Taratima et al. (2020) and Khonghintaisong, Songsri & Jongrungklang (2021) as follows

\begin{eqnarray*}\mathrm{DRI}= \frac{\text{data* of}~\text{DS treatment}}{\text{data of}~\text{FC treatment}} . \end{eqnarray*}

*DRI values demonstrate for traits such as tiller height, tiller number, *P*n, *g*s, *E*, water use efficiency (WUE), SPAD chlorophyll meter reading (SCMR), relative water content (RWC), LT, VBC, CT and SCD.

### Statistical analysis

The measured data were subjected to analysis of variance according to a Factorial in CRD using Statistix 10 (Analytical Software, Tallahassee, FL, USA). Comparisons among genotypes for all data were performed using the Duncan’s Multiple Range Test (DMRT) (Gomez & Gomez, 1984). Correlation analyses among traits were conducted using Pearson’s correlation coefficients.

## Results

### Effect of water regime × genotype on physiological and anatomical traits

An analysis of variance was conducted to assess the relative impacts of water regime (W), genotype (G), and their interaction (W × G) on the variation in morphological, anatomical, and physiological parameters of fifteen sugarcane genotypes under early drought stress (110 DAP). All percentages referred to the percentage of the treatment sum of squares (%S.S.) from Table 1. Morphological traits, including tiller height and tiller number, were highly significantly affected (*P* < 0.01) by genotype, water regime, and their interaction (W × G), and the analysis revealed that the genotype was the most influential factor. Specifically, genotype dominated 59.10% of the variation in tiller height and an even larger proportion in tiller number (76.76%). In contrast, the water regime affected tiller height by 33.01% and tiller number by 14.81%, while the interaction between water regime × genotype (W × G) had a very small effect (Table 1). Across all four anatomical traits (LT, CT, VBC, and SCD), the water regime effect was significant for each trait (*P* < 0.01), reflecting plastic adjustments of leaf anatomy to drought but the effect sizes were relatively small, ranging from 19.28 to 29.27%. The predominance of genotype (G) indicated that anatomical differences among genotypes were largely inherited, overwhelmingly accounting for the majority of variance ranging from 49.67% to 76.75%. The contributions of the interaction between water regime and genotype (W × G) remained small (≤ 3%) and were non-significant for CT, VBC, and SCD (Table 1). For physiological traits, all effects were significant (*P* < 0.01). *P*n, *E*, and *g*s were dominated by the water regime (65.73% to 75.62%), with genotype accounting for 13.01% to 23.39% and the interaction accounted for 8.66% to 10.15. Relative water content (RWC) was significantly influenced by the water regime (47.17%) and genotype (34.15%), whereas their interaction had non-significant effect. For SCMR, the variation attributable to genotype was substantial (41.37%), exceeding that of the water regime, which contributed only 0.96% and was not statistically significant. The genotype × water regime interaction accounted for 12.89% of the total variation, indicating that chlorophyll content remained relatively stable across water treatments but varied among genotypes. Although the water regime (31.31%) had the single largest share of the variance in WUE, the nearly equal contributions from genotype (28.56%) and W × G (29.05%) underscored that both inherent genetic capacity and specific genotype–environment interactions determined WUE. Selection for high WUE therefore needed to consider both average performance and stability across moisture regimes (Table 1). The experiment demonstrated good repeatability, as evidenced by low coefficients of variation for each trait, ranging from 3.74% to 14.42%, except for WUE, which exhibited a relatively high coefficient of variation (Table 1).

**Table 1:** Mean squares for anatomy, growth, and physiology traits of fifteen sugarcane genotypes under early drought conditions at 110 days after planting (DAP).

**SOV**	**Mean squares (%S.S.)**							
	**Water regime (W)**		**Genotype (G)**		**W x G**		**Error**	**CV (%)**
**df**	1		14		14		60	
**Tiller Height**	8,410.0 (33.01)	^**^	1,075.0 (59.10)	^**^	55.8 (3.07)	^**^	20.5 (4.83)	10.09
**Tiller number**	567.5 (14.81)	^**^	210.1 (76.76)	^**^	11.2 (4.11)	^**^	2.8 (4.32)	13.15
**LT**	3,350.7 (18.93)	^**^	970.0 (76.75)	^**^	34.1 (2.70)	^**^	4.8 (1.62)	1.47
**CT**	12.6 (20.40)	^**^	2.3 (51.65)	^**^	0.1 (3.04)	ns	0.3 (24.91)	11.25
**VBC**	1,168.5 (29.27)	^**^	153.6 (53.85)	^**^	8.5 (2.97)	ns	9.3 (13.91)	8.96
**SCD**	103.9 (19.28)	^**^	19.1 (49.67)	^**^	0.7 (1.85)	ns	2.6 (29.20)	14.42
***P*** **n**	3,904.2 (65.73)	^**^	99.2 (23.39)	^**^	36.7 (8.66)	^**^	2.2 (2.22)	5.43
***E***	83.17 (75.62)	^**^	1.02 (13.01)	^**^	0.80 (10.15)	^**^	0.02 (1.22)	8.85
***g*** **s**	0.847 (73.13)	^**^	0.012 (14.26)	^**^	0.008 (9.41)	^**^	0.001 (3.20)	14.89
**WUE**	22,707.4 (31.31)	^**^	1,434.0 (28.56)	^**^	1,458.3 (29.05)	^**^	118.1 (10.08)	37.74
**SCMR**	13.9 (0.96)	ns	43.1 (41.37)	^**^	13.4 (12.89)	ns	10.9 (44.78)	7.84
**RWC**	1,947.1 (47.17)	^**^	100.7 (34.15)	^**^	10.4 (3.54)	ns	10.4 (15.13)	3.74

**Notes.**

* Significant difference at *P* < 0.05.

** Significant difference at *P* < 0.01.

ns Non-significant CV% Coefficient of variation indicated that of the diversity of segregation in each trait % S.S. percentage of sum of squares of segregation in each trait LT Leaf thickness CT cuticle thickness VBC vertical length of bulliform cells SCD stomatal crypt depth Pn net photosynthetic rate E transpiration rate gs stomatal conductance to water vapor WUE water use efficiency SCMR SPAD chlorophyll meter reading RWC relative water content

### Determination of drought effects and DRI on growth in diverse sugarcane genotypes

**Figure 4:**
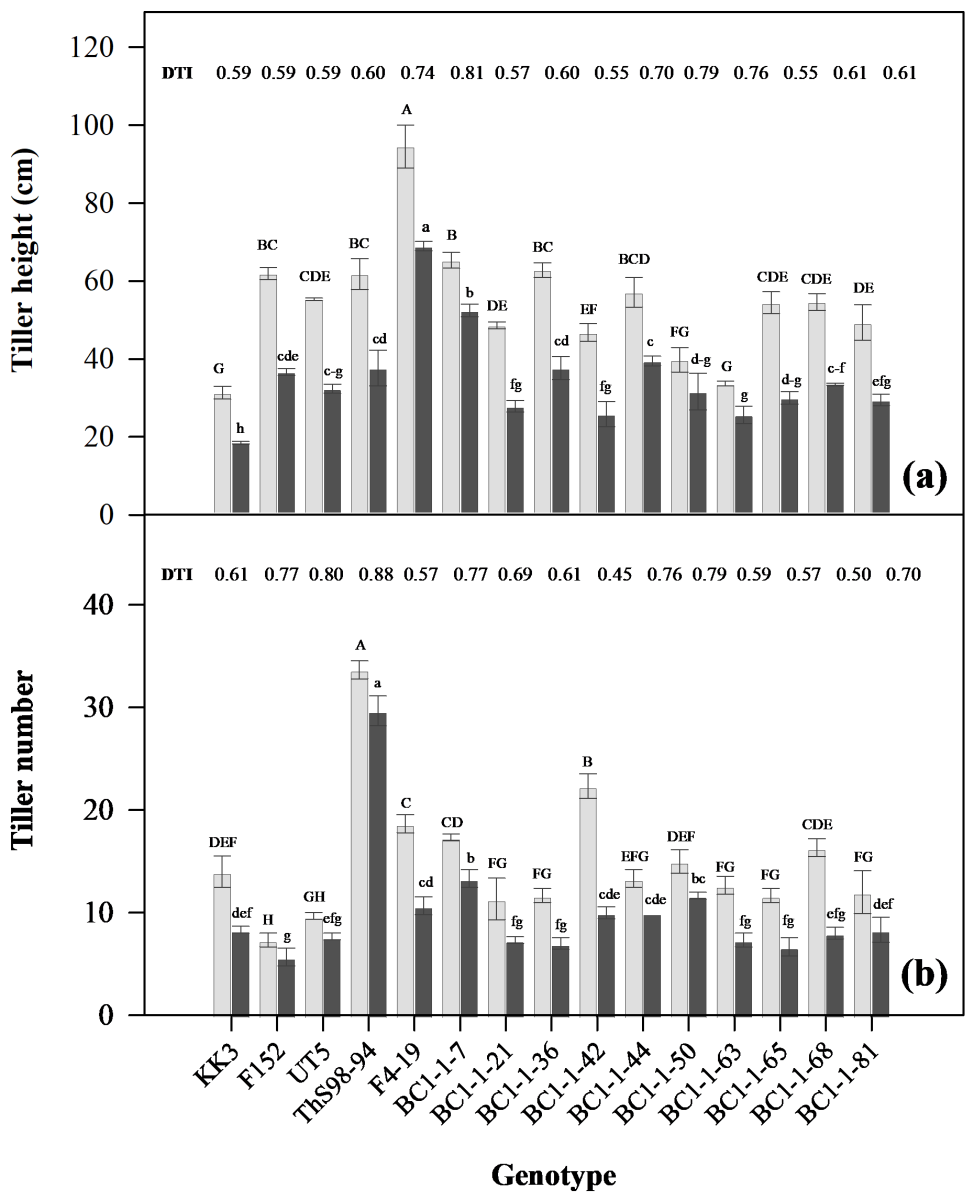
Response of growth traits comprises tiller height (A), tiller number (B) of fifteen sugarcane genotypes under early drought conditions at 110 DAP (days after planting). FC, field capacity (gray bar); DS, drought stress (black bar); and DRI, drought resistance index.

Tiller height decreased for all genotypes under DS compared to FC, indicating inhibited growth due to water deficit. Genotypes BC1-1-7, BC1-1-50, BC1-1-63, F4-19, and BC1-1-44 exhibited higher DRI values of 0.81, 0.79, 0.76, 0.74, and 0.70, respectively, demonstrating better drought resistance (Fig. 4). Conversely, genotypes BC1-1-21, BC1-1-65, and BC1-1-42 had lower DRI values between 0.55 and 0.57, indicating higher susceptibility to drought.

Furthermore, tiller number decreased under DS for most genotypes. Genotypes BC1-1-7, BC1-1-50, BC1-1-44, F152, UT5, and ThS98-94 exhibited higher DRI values for tiller number, ranging from 0.76 to 0.88, indicating better adaptability to drought (Fig. 4). Conversely, genotypes BC1-1-42 and BC1-1-68 had low DRI values of 0.45 and 0.50, respectively, indicating reduced tiller production under drought stress. Overall, genotypes BC1-1-7, BC1-1-44, and BC1-1-50 exhibited higher DRI for both tiller height and tiller number, while BC1-1-42 and BC1-1-65 displayed low DRI for both parameters, indicating limited adaptability to drought (Fig. 4).

### Determination of drought effects and DRI on anatomical traits in diverse sugarcane genotypes

**Figure 5:**
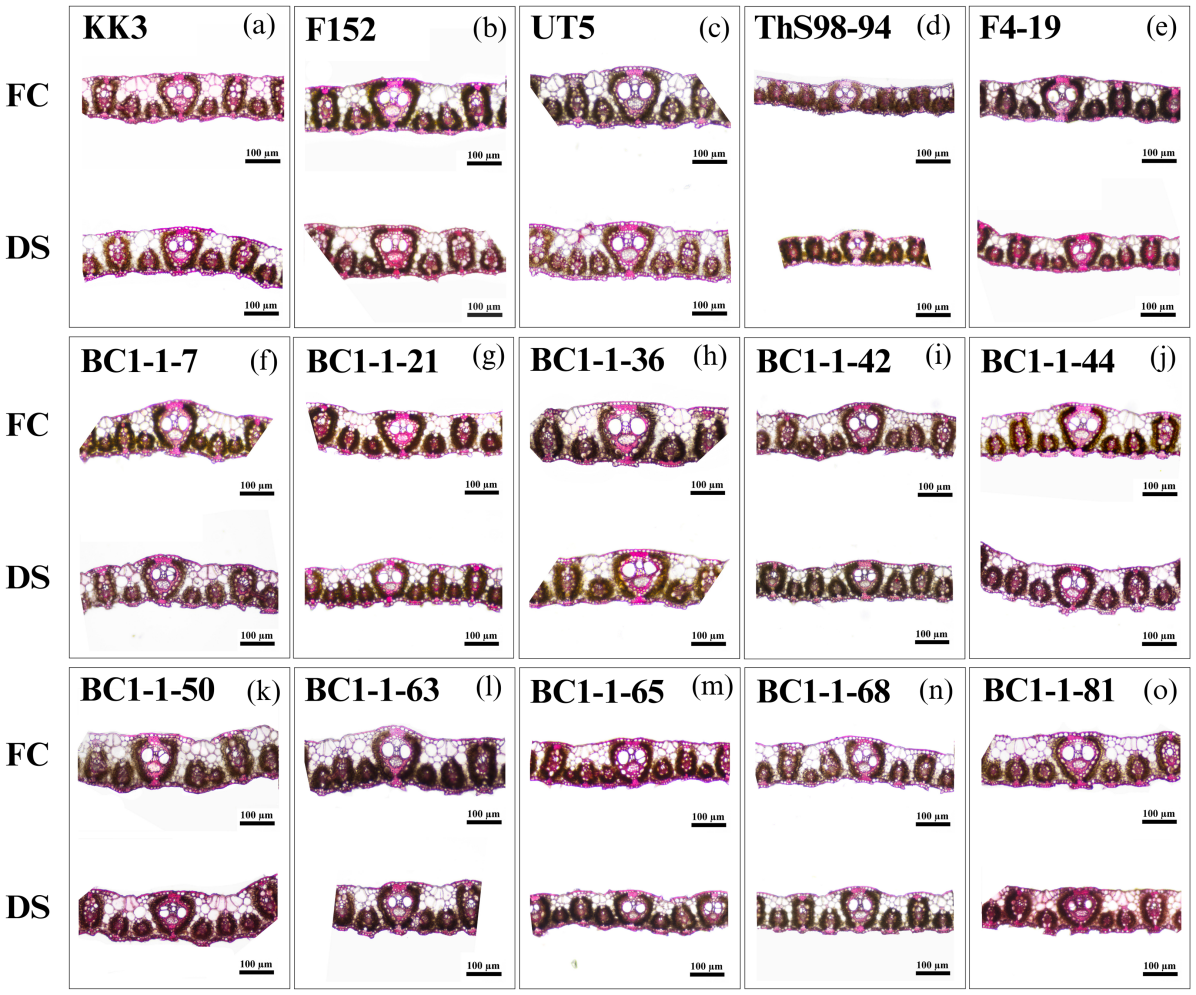
The leaf transverse sections of fifteen sugarcane genotypes were analyzed at 110 days after planting under FC and DS conditions. The leaf transverse sections of fifteen sugarcane genotypes included KK3 (A), F152 (B), UT5 (C), ThS98-94 (D), F4-19 (E), BC1-1-7 (F), BC1-1-21 (G), BC1-1-36 (H), BC1-1-42 (I), BC1-1-44 (J), BC1-1-50 (K), BC1-1-63 (L), BC1-1-65 (M), BC1-1-68 (N), and BC1-1-81 (O) were analyzed at 110 days after planting under field capacity (FC) and drought stress (DS). The scale of each sub–figure is set at 100 µm.

**Figure 6:**
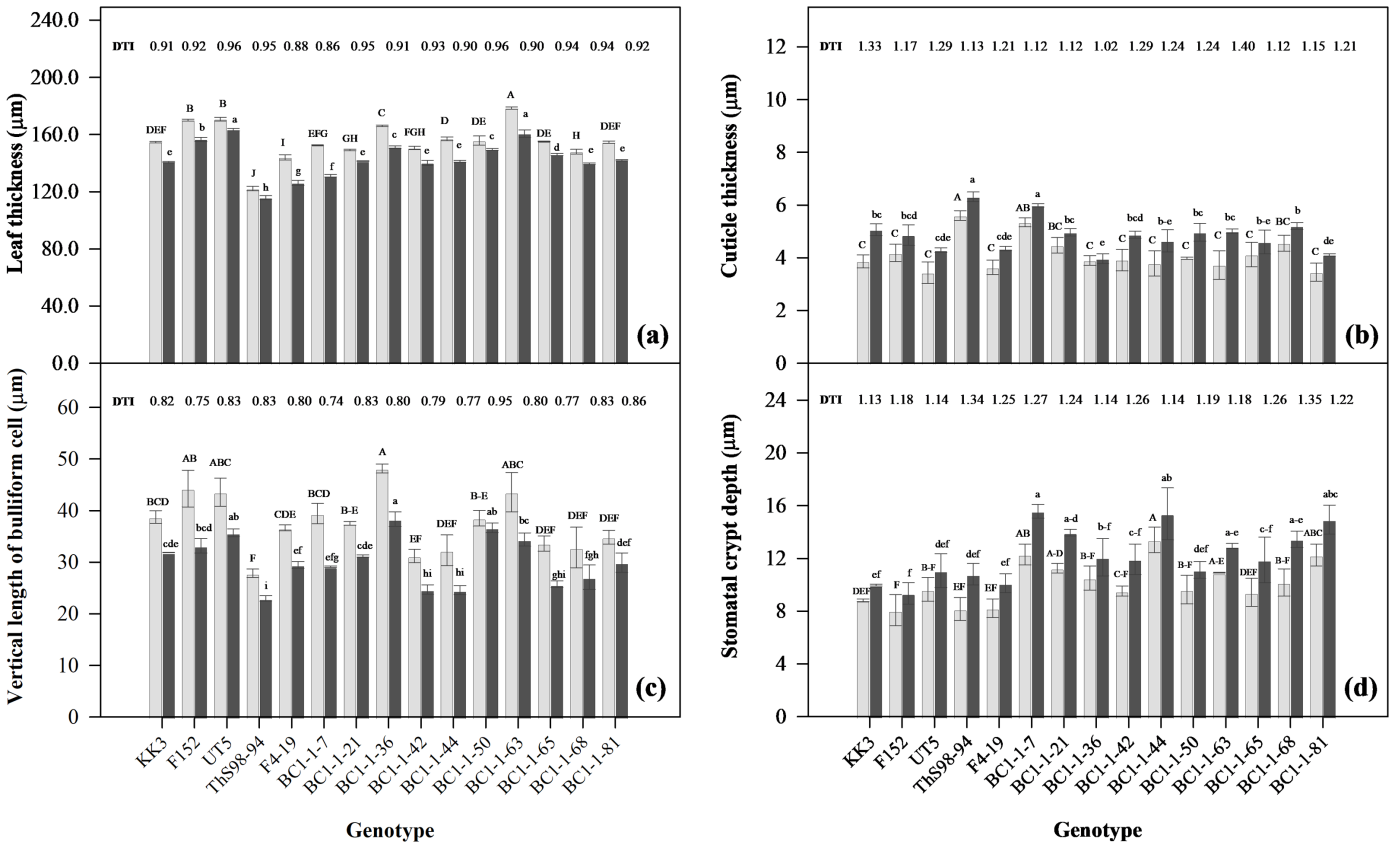
Responses of anatomical traits in fifteen sugarcane genotypes under early drought conditions at 110 days after planting (DAP) under early drought conditions. Responses of anatomical traits; leaf thickness (LT) (A), cuticle thickness (CT) (B), vertical length of bulliform cells (VBC) (C), and stomatal crypt depth (SCD) (D) in fifteen sugarcane genotypes under early drought conditions at 110 days after planting (DAP), FC, field capacity (gray bar) and DS, drought stress (black bar); DRI, drought resistance index.

Fifteen sugarcane genotypes consistently responded to drought stress during early growth stages (Figs. 5 and 6). Water stress significantly reduced LT across all genotypes. Conversely, drought stress increased CT, likely as a protective measure to reduce water loss through transpiration. Additionally, all genotypes exhibited decreased VBC and increased SCD under drought stress, indicating adaptive strategies to cope with limited water availability (Fig. 5). These anatomical changes probably enhance water regulation and minimize water loss during drought conditions.

Under drought stress, BC1-1-7 exhibited reduced LT, a common response among all genotypes. BC1-1-7 showed similarities to F4-19 (male parent) and ThS98-94 (wild type) in traits like low LT and VBC but had a higher cuticle thickness (6.0 µm). Additionally, BC1-1-7 had a high SCD (15.6 µm), similar to BC1-1-44 (15.4 µm) and BC1-1-81 (14.9 µm), compared to F4-19 (10.1 µm) and ThS98-94 (10.8 µm). BC1-1-44 demonstrated more effective anatomical adaptations to drought stress than BC1-1-50, with low potential and DRI for LT and VBC and high SCD potential but low DRI, indicating enhanced water conservation mechanisms. These variations in anatomical traits under drought stress can be attributed to genetic inheritance from their parent plants. BC1-1-7 and BC1-1-44 showed similar anatomical characteristics to their male parent (F4-19) in terms of LT and VBC, with low potential and DRI values but high potential for SCD compared to F4-19. Conversely, BC1-1-50 showed responses similar to its female parent (UT5) for LT and VBC, with high potential and DRI values, while the SCD trait showed low potential and DRI (Fig. 6). The BC1 interspecific hybrid genotypes BC1-1-7, BC1-1-50, and BC1-1-44 exhibited distinct anatomical responses under drought stress. BC1-1-7 and BC1-1-44 were similar to F4-19 (male parent) in LT, CT, and VBC, with BC1-1-44 displaying high potential for SCD. Both BC1-1-7 and BC1-1-44 had favorable responses to drought stress, while BC1-1-50 demonstrated effective anatomical adaptations similar to UT5 (female parent) in LT, CT, VBC, and SCD.

### Determination of drought effects and DRI on physiological traits in diverse sugarcane genotypes

**Figure 7:**
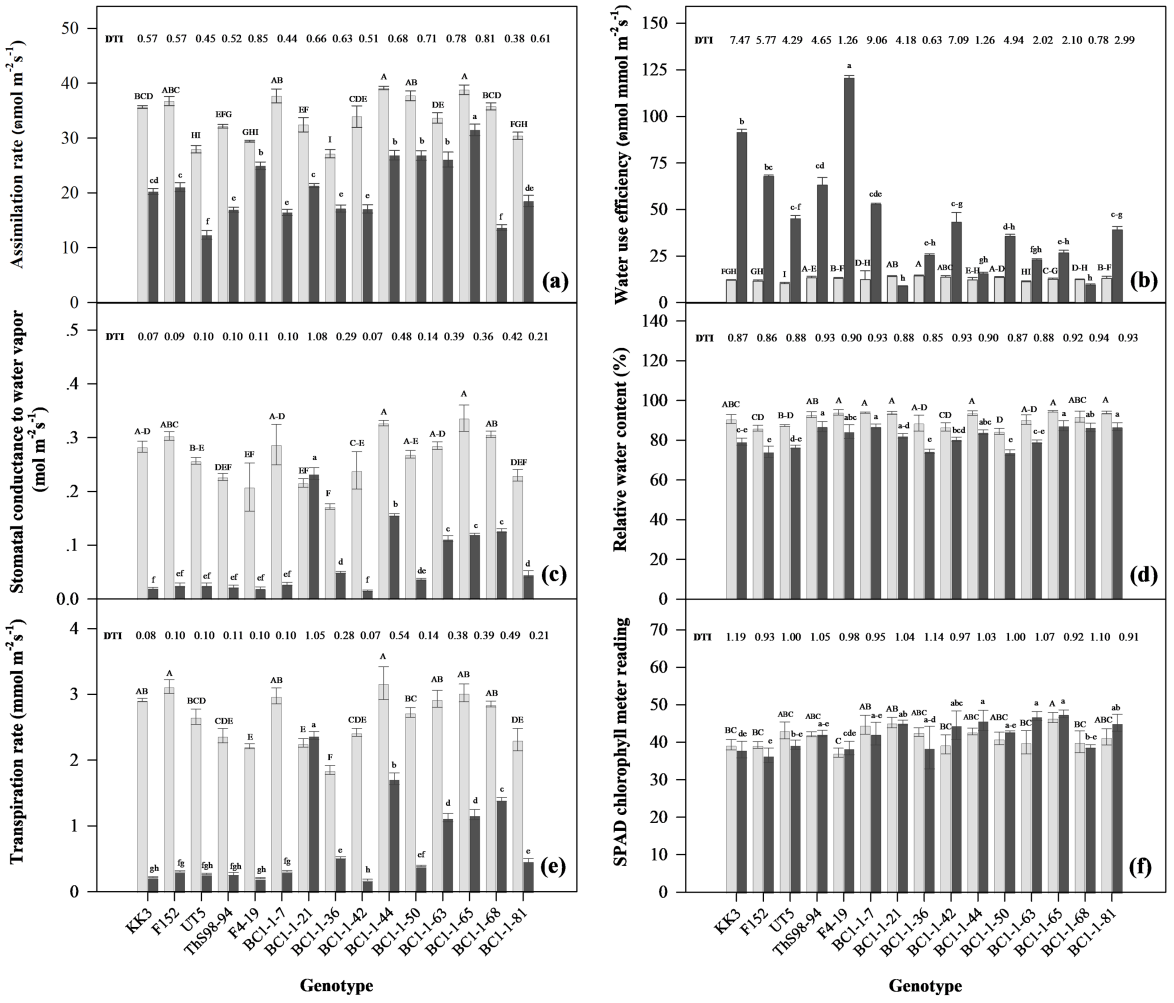
Response of physiological traits of fifteen sugarcane genotypes under early drought conditions at 110 days after planting (DAP). Response of physiological traits; net photosynthetic rate (*P*_*n*_) (A), water use efficiency (WUE) (B), stomatal conductance to water vapor (*g*_*s*_) (C), relative water contents (RWC) (D), transpiration rate (*E*) (E), and SPAD chlorophyll meter reading (SCMR) (F) of fifteen sugarcane genotypes under early drought conditions at 110 days after planting; FC, field capacity (gray bar) and DS, drought stress (black bar); DRI, drought resistance index.

The genotypes displayed distinct physiological responses to drought stress. BC1-1-7, ThS98-94, and BC1-1-81 struggled to maintain assimilation, showing relatively low DRI for traits such as *P*n, *g*s, *E*, and SCMR. However, they exhibited high DRI for WUE and RWC. BC1-1-7 performed well in *g*s, *E*, WUE, and RWC, comparable to F4-19 (male parent), ThS98-94, and BC1-1-81. BC1-1-44 demonstrated exceptional physiological responses, showing high DRI for *P*n, *g*s, *E*, and SCMR, similar to BC1-1-21 and BC1-1-65, although BC1-1-65 had a low DRI for SCMR (Fig. 7). The response of BC1-1-50 to drought stress mirrored that of commercial genotypes like F4-19 and KK3, with F4-19 and KK3 exhibiting high WUE potential, while both BC1-1-50 and F4-19 had high *P*n potential.

BC1-1-44 and BC1-1-50 revealed the highest *P*n under both FC and DS conditions, similar to the male parent F4-19, while BC1-1-7 showed the lowest *P*n under drought stress. BC1-1-44 also had higher *g*s under both conditions compared to BC1-1-7 and BC1-1-50. However, BC1-1-7 and BC1-1-50 demonstrated higher WUE under drought stress, similar to F4-19, ThS98-94, KK3, and F152, indicating better adaptation to limited water availability. In terms of SCMR, BC1-1-7, BC1-1-44, and BC1-1-50 exhibited high DRI and only a slight decrease under drought stress, suggesting more effective chlorophyll synthesis and photosynthetic capacity under stress conditions. Regarding RWC, BC1-1-50 showed the highest decrease under drought stress, indicating greater water loss compared to BC1-1-7 and BC1-1-44. Overall, BC1-1-7 and BC1-1-50 exhibited greater resistance to drought stress for physiological parameters such as low *g*s, *E*, and high WUE compared to BC1-1-44. BC1-1-44 showed a specific response of high *P*n, *g*s, *E*, and SCMR under drought stress, indicating potential adaptive mechanisms for water use and photosynthesis.

### Correlation of anatomical and physiological responses related to drought resistance

The LT have a significant negative correlation (*P* <0.01) with tiller number under FC and DS (Table 2), indicating that thicker leaves might reduce the number of tillers. In addition, the VBC exhibits a significant negative correlation (*P* < 0.01) with tiller number under FC (Table 2). On the contrary, the CT has significant*P* < 0.01 and *P* < 0.05 positive correlations with tiller number under FC and DS, respectively (Table 2), implying that thicker cuticles may enhance tiller number. The LT significant negative correlation (*P* < 0.05) with CT and significant positive correlation (*P* < 0.01) with VBC under both FC and DS (Table 2), suggesting a role in leaf rolling for water conservation. However, LT and VBC was strongly significant negative correlation (*P* < 0.01) with RWC under FC and DS (Table 2), highlighting a trade-off between structural adaptation and water retention. The SCD exhibits significant positive correlation (*P* < 0.05) with *g*s*, E*, and SCMRbut exhibits significant negative correlation (*P* < 0.05) with WUE under DS (Table 2), indicating that under drought stress, sugarcane genotypes with greater stomatal crypt depth tend to have higher transpiration rates and stomatal conductance, meaning that these deeper crypts are associated with increased water loss. The *P*n exhibits significant positive correlation (*P* < 0.05) with *g*s and *E* under FC (Table 2). In other words, plants that maintain higher *g*s allow more CO_2_ to enter the leaves, which in turn supports increased photosynthesis while also exhibiting higher water loss through transpiration. The *g*s exhibits significant positive correlation (*P* < 0.01) with *E* under FC and DS but exhibits significant negative correlation with WUE under FC (*P* < 0.05) and DS (*P* < 0.01) (Table 2). The *E* exhibits significant negative correlation (*P* < 0.01) with WUE under FC and DS (Table 2).

**Table 2:** Correlations among anatomical traits, growth traits and physiological traits in fifteen sugarcane genotypes under early drought stress at 110 days after planting (DAP).

	**Tiller Height**	**Tiller number**	**LT**	**CT**	**VBC**	**SCD**	***P*** **n**	***g*** **s**	***E***	**WUE**	**SCMR**
**Field Capacity (FC)**											
**Tiller number**	0.199										
**LT**	−0.323	−0.826^**^									
**CT**	0.155	0.605^*^	−0.618^*^								
**VBC**	−0.054	−0.673^**^	0.794^**^	−0.355							
**SCD**	−0.181	−0.279	0.217	−0.076	−0.013						
***P*** **n**	−0.292	−0.047	−0.033	0.292	−0.331	0.166					
***g*** **s**	−0.288	−0.255	0.226	0.057	−0.192	0.141	0.847^**^				
***E***	−0.300	−0.260	0.290	0.078	−0.073	0.118	0.827^**^	0.966^**^			
**WUE**	0.145	0.351	−0.504	0.234	−0.256	0.048	−0.142	−0.587^*^	−0.671^**^		
**SCMR**	−0.096	−0.166	−0.019	0.315	−0.034	0.446	0.178	0.170	0.083	0.104	
**RWC**	0.320	0.181	−0.437	0.264	−0.445	0.378	0.060	0.075	−0.014	0.102	0.355
**Drought Stress (DS)**											
**Tiller number**	0.224										
**LT**	−0.459	−0.728^**^									
**CT**	0.029	0.710^**^	−0.549^*^								
**VBC**	−0.116	−0.471	0.688^**^	−0.441							
**SCD**	0.012	−0.085	−0.096	0.056	−0.253						
***P*** **n**	0.037	−0.179	0.083	−0.167	−0.065	−0.047					
***g*** **s**	−0.227	−0.286	0.115	−0.056	−0.152	0.519^*^	0.326				
***E***	−0.216	−0.273	0.100	−0.041	−0.171	0.531^*^	0.305	0.998^**^			
**WUE**	0.331	0.226	−0.329	0.049	−0.032	−0.631^*^	−0.025	−0.770^**^	−0.775^**^
**SCMR**	−0.283	−0.007	0.060	−0.077	−0.225	0.584^*^	0.462	0.506	0.480	−0.487	
**RWC**	0.256	0.358	−0.680^**^	0.351	−0.798^**^	0.510	0.023	0.252	0.257	−0.181	0.293

**Notes.**

* Significant difference at *P* < 0.05.

** Significant difference at *P* < 0.01.

ns Non-significant LT Leaf thickness CT cuticle thickness VBC vertical length of bulliform cells SCD stomatal crypt depth Pn net photosynthetic rate E transpiration rate gs stomatal conductance to water vapor WUE water use efficiency SCMR SPAD chlorophyll meter reading RWC relative water content

## Discussion

Drought is a major factor limiting agricultural production, significantly impacting crop yields (Begna, 2020). One of the important findings of this study was that neither the water regime nor the genotype had a significant effect on SCMR (Table 1). The water regime affected various parameters, except for the SPAD chlorophyll meter reading (SCMR), as drought-tolerant cultivars exhibited minimal changes in chlorophyll content (da Silva et al., 2018; Nascimento & Marenco, 2010). However, the genotype had a greater impact on the variance than the water regime, indicating that the photosynthesis was maintained to some extent even under dry conditions. This suggested that the plants did not experience severe stress, which would normally cause considerable chlorophyll degradation, a symptom of severe drought stress. Variable sensitivity to drought among different genotypes has been linked to distinct physiological responses depending on the duration of drought treatments (Tippayawat et al., 2023). The minor influence of drought on chlorophyll concentration in this study could be attributed to the short duration and mild nature of the drought stress administered. In some genotypes, *P*n remained relatively high, such as in BC1-1-44, BC1-1-50, BC1-1-63, and BC1-1-65 (Fig. 7). Some hybrids demonstrated greater resistance by maintaining higher photosynthesis levels under water-deficient conditions, highlighting the importance of physiological responses in drought tolerance (Tippayawat et al., 2023). Both genotype and water regime affected *P*n, but the water regime had a greater effect (Table 1). This implied that certain genotypes were able to modify physiological functions to maximize CO_2_ uptake and reduce water loss. The relatively mild stress in the present study was likely due to the drought duration and the use of large pots, which allowed more extensive root development, enabling plants to access a greater volume of soil and, consequently, more water and nutrients. This could have mitigated the effects of drought stress by supporting water uptake and maintaining physiological processes such as photosynthesis. The influence of container size and depth on root development and plant drought responses has been extensively studied. A meta-analysis by Poorter et al. (2012) found that doubling pot size led to an average increase in plant biomass of 43%, with growth enhancement primarily attributed to increased photosynthesis per unit leaf area rather than changes in leaf morphology or biomass allocation. Moreover, genotypic differences in root length density and distribution can affect water uptake efficiency; for example, certain genotypes exhibit higher root length density in upper soil layers, which can be advantageous under specific drought scenarios (Chumphu, Jongrungklang & Songsri, 2019). In this study, the water regime accounted for only a small proportion of the total variation in anatomical traits, while genotypic differences were the dominant source of variation. This suggests that most anatomical features were already established before the onset of drought stress, limiting the extent of plastic anatomical responses during the experimental period. Therefore, the results indicate that genotypes explained more of the variation in anatomical traits than water regime effects, rather than implying that drought stress had no influence on these traits. The limited effect of water regime on anatomical traits, such as leaf structure and stomatal density, aligns with studies indicating that these traits are predominantly determined by genetic factors. For example, in oak trees, leaf anatomical characteristics associated with water use efficiency were found to be more influenced by genotype than by environmental conditions (Roussel et al., 2009). Furthermore, research on sorghum revealed that while hydraulic anatomy can affect plant water use, genotypic variation in xylem anatomy showed differential plasticity to drought, with certain genotypes exhibiting greater anatomical adjustments under water stress (Guha et al., 2018). This study revealed a complex interplay between genetic control and environmental factors in determining sugarcane traits under drought stress. Different sugarcane genotypes exhibited varying physiological responses to drought, influencing photosynthesis and biomass yield (Tippayawat et al., 2023). In this study, genotypes were the most significant contributor to variation in morphology (tiller height and tiller number), accounting for over 50% of the observed variation, as well as anatomical traits such as LT, CT, VBC, and SCD, which were also predominantly influenced by genotype, with contributions ranging from 49.67% to 76.75% (Table 1). Meena et al. (2020) reported that key shoot parameters, including stalk height, tiller population, stalk diameter, stalk weight, and the number of millable shoots, were more variable and genotype-dependent under drought stress conditions. Similarly, Wiangwiset et al. (2023a) and Wiangwiset et al. (2023b) found significant genotypic diversity in anatomical characteristics of sugarcane, including leaf thickness, cuticle thickness, vertical bulliform cell percentage, and stomatal crypt depth in commercial, wild-type, and F1 interspecific hybrids. The strong genetic control of these traits suggests that selection for improved drought resistance through breeding can be effectively achieved by focusing on genotype-specific characteristics. The water regime predominantly influenced physiological traits such as *P*n, *E*, and *g*s, with water availability accounting for 65.73–75.62% of the variation in these processes (Table 1). This indicates that water stress immediately affected plant function. Under drought stress, reduced water availability limited stomatal opening and gas exchange in sugarcane, thereby reducing photosynthetic, while water use efficiency increased (Leanasawat et al., 2021). This study demonstrated a notable interaction between genotype and water regime, with nearly equal contributions from both factors (31.31% for water regime, 28.56% for genotype, and 29.05% for W × G) (Table 1). This complex interaction for WUE illustrates the vital roles of both environmental adaptation and genetic potential in sugarcane’s drought response. The percentage contribution of each source (%S.S.) revealed the factors most responsible for variation in individual traits. Morphological (tiller number/height) and anatomical (LT, CT, VBC, SCD) traits were under strong genetic control, suggesting high heritability and amenability to direct selection. Physiological traits (*P*n, *E*, *g*_*s*_, RWC) were predominantly influenced by water regime, underscoring the immediate impact of drought stress on plant function. Traits with substantial W × G interaction (notably WUE) may require evaluation of specific genotype performance under varying water regimes to identify candidates with superior combined responsiveness. These results underscore the differential responses of various genotypes to changes in water regime, highlighting genetic variability in their capacity to adapt to drought. To identify superior genotypes with resilient drought resistance, performance under both normal and drought conditions must be carefully assessed. Correlation analysis in this study revealed intricate relationships among morphological, physiological, and anatomical traits under drought stress (Table 2). The positive correlation between *P*n and *E* and *g*s (Table 2) suggested that plants with higher stomatal conductance and transpiration rates could support higher CO_2_ fixation and increased photosynthesis, which were critical for growth. Silva et al. (2013) found that the TCP02-4587 genotype exhibited higher transpiration and photosynthesis rates, indicating effective stomatal control, and this positive correlation between photosynthesis, stomatal conductance, and transpiration rate highlights its adaptation to water deficit. From the previous study of Lawlor & Tezara (2009), plants close their stomata during water shortages to maintain turgor pressure, a key trait for drought resistance. However, these physiological functions are energetically demanding and can increase water loss, underscoring the need to balancing photosynthetic efficiency with water conservation. Similarly, the positive correlation between SCD and both *E* and *g*s suggests the role of deeper crypts in maintaining stomatal function (Table 2). However, these deeper crypts also lead to higher water loss, further emphasizing the need to balance gas exchange for CO_2_ uptake (carbon gain) with water conservation. The negative correlation with WUE highlights this trade-off. The relationship between deeper crypts and higher water loss—and the need to balance gas exchange with water conservation—is complex and influenced by factors such as species adaptation to dry or wet climates and the effects of water stress on photosynthesis (Manzoni et al., 2011). Marginal WUE can vary across plant functional types and climates, with different responses to soil water availability affecting the optimization of stomatal conductance for maximum carbon gain (Manzoni et al., 2011). Anatomical traits such as LT and VBC also showed a negative correlation with tiller number and RWC (Table 2), suggesting that while thicker leaves and increased VBC may enhance structural integrity and support protective mechanisms such as leaf rolling, this comes at the cost of reduced RWC, as the plant sacrifices water retention to develop more robust vascular structures, which can limit growth and reduce the plant’s ability to stay hydrated when water is scarce. Singh, Singh & Chauhan (2011) reported that in rice leaves, the degree of leaf rolling was positively correlated with the rate of water loss; partial rolling reduces water loss by 36%, while complete rolling reduced it by 52%. Nevertheless, depending on the rice variety, the response may be mediated by several different mechanisms or reactions, making it impossible to predict drought performance based solely on leaf rolling (Gunnula et al., 2022). These results underscore that improvements in one trait can influence others, highlighting the importance of integrative trait selection in developing drought-resilient plant varieties.

Comparative transcriptome analysis revealed significant upregulation of stress resistance genes and synthesis pathways in hybrids compared to their parents (Feng et al., 2023). This study investigated the growth, anatomical, and physiological responses to drought stress in BC1 progeny resulting from interspecific hybridization between *Saccharum* spp. hybrids and *S. spontaneum*. The findings highlighted significant genetic variability among the progeny, showing distinct variations in leaf anatomical structures, growth patterns, and physiological processes, which played pivotal roles in drought adaptation. Notably, certain BC1 progeny demonstrated enhanced leaf thickness, improved stomatal conductance, and superior water use efficiency, indicating their potential for superior drought resistance. Features of crossing parents (ThS98-94, F152, F4-19, UT5): ThS98-94 (wild-type), this genotype was characterized by strong anatomical traits such as increased CT and SCD (Fig. 6), possibly due to these structural adaptations that helped conserve water. *S. spontaneum* demonstrated high yield, early vigor, ratooning ability, low input requirements, and tolerance to various stresses, making it advantageous for biomass production under challenging conditions (Silva, Pincelli & Barbosa, 2018). However, ThS98-94 showed low physiological stability under drought stress, particularly for *P*n and *g*s, although it maintained a high WUE (Fig. 7). ThS98-94 may have used strategies to minimize water loss, such as decreased transpiration rates or osmotic adjustments, to maintain high WUE under drought stress. Certain sugarcane genotypes maintained higher WUE under drought stress, but this was not always accompanied by higher *P*n, indicating that WUE could be influenced by factors other than photosynthetic capacity (Khonghintaisong et al., 2024). Drought lowers both photosynthesis and transpiration, but WUE increases when transpiration declines more than photosynthesis. For example, in the study of Khonghintaisong et al. (2024), drought-resistant genotypes such as KK3 and UT13 demonstrated outstanding WUE but did not show increasing net photosynthesis and transpiration rates under rainfed conditions during drought and recovery periods. This suggested that high WUE could result from reduced water loss rather than increased carbon assimilation. F152 was a commercial genotype with moderate drought resistance for anatomical traits CT and SCD, enabling it to handle some drought stress, though not as well as wild type (Fig. 6). In addition, F152 maintained high *P*n and WUE under drought conditions, making it well-suited for dry environments, though it showed a drop in physiological parameters like *g*s and *E* (Fig. 7). Considering its inheritance, F152 was likely to pass on its high *P*n and WUE traits to its hybrid, as evidenced by the findings in F4-19. F4-19 (F1 interspecific hybrid between ThS98-94 x F152), as a male parent, was a key genotype for drought resistance; it demonstrated high WUE and *P*n (Fig. 7), and its anatomical traits, such as lower LT and VBC under drought conditions, were likely to contribute to its enhanced drought resistance (Fig. 6). UT5 exhibited moderate cuticle thickness and stomatal density under stress, suggesting some level of drought adaptation (Fig. 6). In terms of physiological traits, UT5 (commercial parent) demonstrated greater responsiveness to water conservation, sustaining moderate levels of *P*n, *g*s, and WUE during drought conditions (Fig. 7). These traits were passed down to its BC1 hybrid, like BC1-1-7 and BC1-1-44, which demonstrated improved drought resistance. This study revealed diverse abilities of BC1 progeny from interspecific hybridization between *Saccharum* spp. Hybrids and *S. spontaneum* to adapt to drought stress, primarily through variations in leaf anatomy, growth, and physiological processes. The observed diversity could be attributed to the distinct genetic backgrounds of the progeny, which inherited different traits from the parent species UT5 (female parent) and F4-19 (male parent). Specifically, the BC1 hybrid displayed a variety of leaf anatomical adaptations that minimized water loss during drought conditions, such as thicker CT, and reduced LT (Fig. 6). Interspecific hybridization between *Saccharum* spp. hybrids and *S. spontaneum* clones in various populations produced backcross (BC1) progeny with distinct anatomical characteristics, including stomatal crypt depths, cuticle thickness, and bulliform cells, which influenced their potential for yield and drought tolerance (Wiangwiset et al., 2023a; Wiangwiset et al., 2023b). Furthermore, certain hybrid showed improved physiological responses, like increased net photosynthetic capacity, water use efficiency, and transpiration rate, which enabled them to stay hydrated during water shortages (Fig. 7). Overall, BC1 clones inherited a complex of traits from their parental lines through backcrossing, with each parent contributing both anatomical and physiological characteristics that helped the progeny adapt to drought stress. The integration of these traits, such as high WUE, *P*n, and SCD, alongside favorable anatomical characteristics like increased CT and lower LT in BC1 genotypes suggested a degree of genotypic diversity in drought responses, with some clones demonstrating higher drought resistance (Type I) while others showed weaker responses (Type IV). These characteristics collectively contributed to the drought resistance of interspecific hybrid sugarcane. In this study, F1 interspecific hybrids exhibited the greatest tiller height, followed by BC1 interspecific hybrids. These traits were likely influenced by the genetics of the wild type *S. spontaneum*, known for its adaptability to various environments. The wild type demonstrated the highest number of shoots under both fully watered and drought conditions compared to other genotypes. In addition, *S. spontaneum* exhibited diverse morphological adaptations, including plant height, tillering capacity, stalk diameter, internode length, biomass potential, and stress resistance (Govindaraj & Amalraj, 2022). Leveraging the genetic diversity found in wild sugarcane germplasm could enhance these traits in commercial varieties, ensuring better yields and resistance in changing environmental conditions (Govindaraj & Amalraj, 2022). Under drought stress conditions, most BC1 interspecific hybrid genotypes in this study showed better tiller number responses compared to commercial varieties. BC1 interspecific hybrids exhibited a higher number of tillers compared to the female parent, suggesting an enhanced ability to grow, especially in terms of tiller production. Tillering significantly affected yield variability by increasing the number of stalks, enhancing photosynthetic capacity, influencing resource allocation, and providing adaptability to environmental conditions (Govindaraj & Amalraj, 2022).

A key response in leaf anatomical structure under drought stress across all genotypes was significantly reduced leaf thickness and increased cuticle thickness, a crucial drought tolerance indicator (Taratima et al., 2019; Taratima et al., 2020). Drought stress significantly impacts leaf thickness in sugarcane, affecting its growth and physiological responses. It altered leaf anatomy and cellular structures, which were crucial for maintaining plant health under water deficit conditions. Under drought, leaf thickness often decreased due to reduced leaf area and surface expansion rates, as observed in tobacco (Khan et al., 2023). Increased cuticle thickness served as a natural barrier, preventing excessive water loss through transpiration and helping the plant retain hydration during periods of water scarcity (Valarmathi et al., 2022). This anatomical adaptation, along with traits such as leaf epidermal thickness and intact bulliform cells, enhanceed the plant’s ability to cope with drought (Valarmathi et al., 2022). The vertical length of bulliform cells was particularly important as these cells facilitated leaf rolling, minimizing sunlight exposure and reducing water loss during water stress (Valarmathi et al., 2022). Moreover, deeper stomatal crypts regulated gas exchange and further reduced water loss, enabling the plant to maintain photosynthetic efficiency under drought conditions (Valarmathi et al., 2022). Overall, these anatomical adaptations worked synergistically to improve water retention and stress resistance, making them vital for the survival and productivity of sugarcane in arid environments (Masoabi et al., 2023; Wirojsirasak et al., 2024; Hoffman, Singels & Joshi, 2024). Furthermore, reduced vertical length of bulliform cells and increased stomatal crypt depth could have significantly affected photosynthesis and transpiration rates, impacting gas exchange (Harrison et al., 2020). The leaf anatomical traits of the BC1 interspecific hybrids in this study exhibited diverse responses to drought across different genotypes. Observed adaptations in the wild-type species included reduced leaf thickness, smaller bulliform cells, increased cuticle thickness, and deeper stomatal crypts, which contributed to drought resistance. Wild sugarcane was characterized by low leaf thickness, but high stomatal crypt depth and cuticle thickness compared to commercial varieties, enhancing its adaptability to drought conditions (Wiangwiset et al., 2023a; Wiangwiset et al., 2023b). These variations in the anatomical traits of sugarcane interspecific hybrids under drought stress could be attributed to genetic inheritance from their parent plants (Taratima et al., 2020). The parental lines, particularly those originating from drought-tolerant species such as *S. spontaneum*, likely contributed to adaptive traits that enhanced the hybrid ability to cope with water-limited conditions (Alarmelu et al., 2025). For example, traits such as reduced leaf thickness, thicker leaf cuticles, and deeply sunk stomata crypt were observed (Jumkudling et al., 2022). This highlighted the critical role of selective breeding in transferring advantageous traits to progeny. These variations indicated genetic diversity in coping with water deficits, highlighting the potential for developing drought-resistant sugarcane varieties.

Drought stress in sugarcane primarily induces stomatal closure to reduce water loss, which consequently limits CO_2_ uptake and reduces photosynthetic rate (Yang et al., 2023). This decline in photosynthesis may be accompanied by reduced chlorophyll content and nutrient transport, leading to lower rubisco activity and overall photosynthetic efficiency (Yang et al., 2023). In sugarcane, these physiological constraints ultimately contribute to reduced growth and biomass accumulation (Yang et al., 2023; Khonghintaisong, Songsri & Jongrungklang, 2021). Distinct physiological responses to drought stress were observed in different genotypes, affecting photosynthetic rate, stomatal conductance, transpiration, water use efficiency, and relative water content. These results align with Misra, Mall & Pathak (2022), who described genotype-specific drought avoidance and tolerance strategies in sugarcane. Genotypes that reduced stomatal conductance and transpiration maintained higher RWC, reflecting an avoidance strategy, while those sustaining photosynthesis and WUE under stress demonstrated tolerance mechanisms (Misra, Mall & Pathak, 2022). Such variation highlights the trade-off between carbon gain and water conservation that underpins genotypic differences in drought adaptation. Interspecific sugarcane hybrids displayed varying sensitivities to drought in physiological traits, with each genotype responding uniquely to different drought durations (Tippayawat et al., 2023). Some sugarcane genotypes in this study exhibited higher transpiration rates and improved water use efficiency under stress conditions. F4-19, as the F1 interspecific hybrid, was developed by crossing a wild type Ths98-94 with F152 commercial sugarcane and KK3, a commercial drought-resistant variety, both of which exhibited high WUE potential. Both genotypes potentially possessed traits such as efficient stomatal regulation and optimized leaf anatomical structures, allowing them to perform well despite limited water availability. F4-19, an F1 interspecific hybrid, appeared to contribute traits such as enhanced net photosynthetic (*P*n) capacity and efficient stomatal regulation, which supported high WUE. KK3, as a commercial drought-resistant check, was known for its effective water management under stress. KK3′s high WUE could have stemmed from its ability to balance stomatal closure and maintain photosynthesis under stress, reducing water loss while sustaining productivity. Moreover, the similar responses observed in BC1-1-50 and F4-19 regarding the high net photosynthetic rate (*P*n) potential could be attributed to their intrinsic ability to conduct photosynthesis effectively even under drought stress. F4-19, being a hybrid, likely inherited beneficial traits from both its wild type, ThS98-94, and commercial parent, F152, due to both varieties displaying high WUE values in this investigation. Additionally, BC1-1-50, being a second-generation hybrid of F4-19 crossed with a commercial variety, may have retained or even enhanced these advantageous traits, improving its drought performance. These findings underscored the importance of interspecific hybridization in combining favorable traits from diverse parental lines to enhance drought resilience in sugarcane. The response of sugarcane to drought stress, particularly its impact on transpiration rate, growth, and yield, was influenced by factors such as growth stage, genotype, and drought severity (Eksteen & Singels, 2014; dos Santos et al., 2019). Wild-type sugarcane (*S. spontaneum*) demonstrated various physiological adaptations that supported robust growth and biomass production in drought conditions. These adaptations included deep root growth for enhanced water uptake, reduced stomatal conductance, osmotic adjustment, and ROS detoxification (Dhansu et al., 2022). Moreover, it maintained high levels of photosynthesis and stomatal conductance under moderate stress (Masoabi et al., 2023) and exhibited stay-green traits, such as a higher chlorophyll fluorescence ratio, leaf chlorophyll content, and relative water content, contributing to drought tolerance (Wirojsirasak et al., 2024). Furthermore, wild-type sugarcane showed variable sensitivity to drought among genotypes, with some maintaining relatively higher photosynthetic activity under stress conditions (Tippayawat et al., 2023).

**Figure 8:**
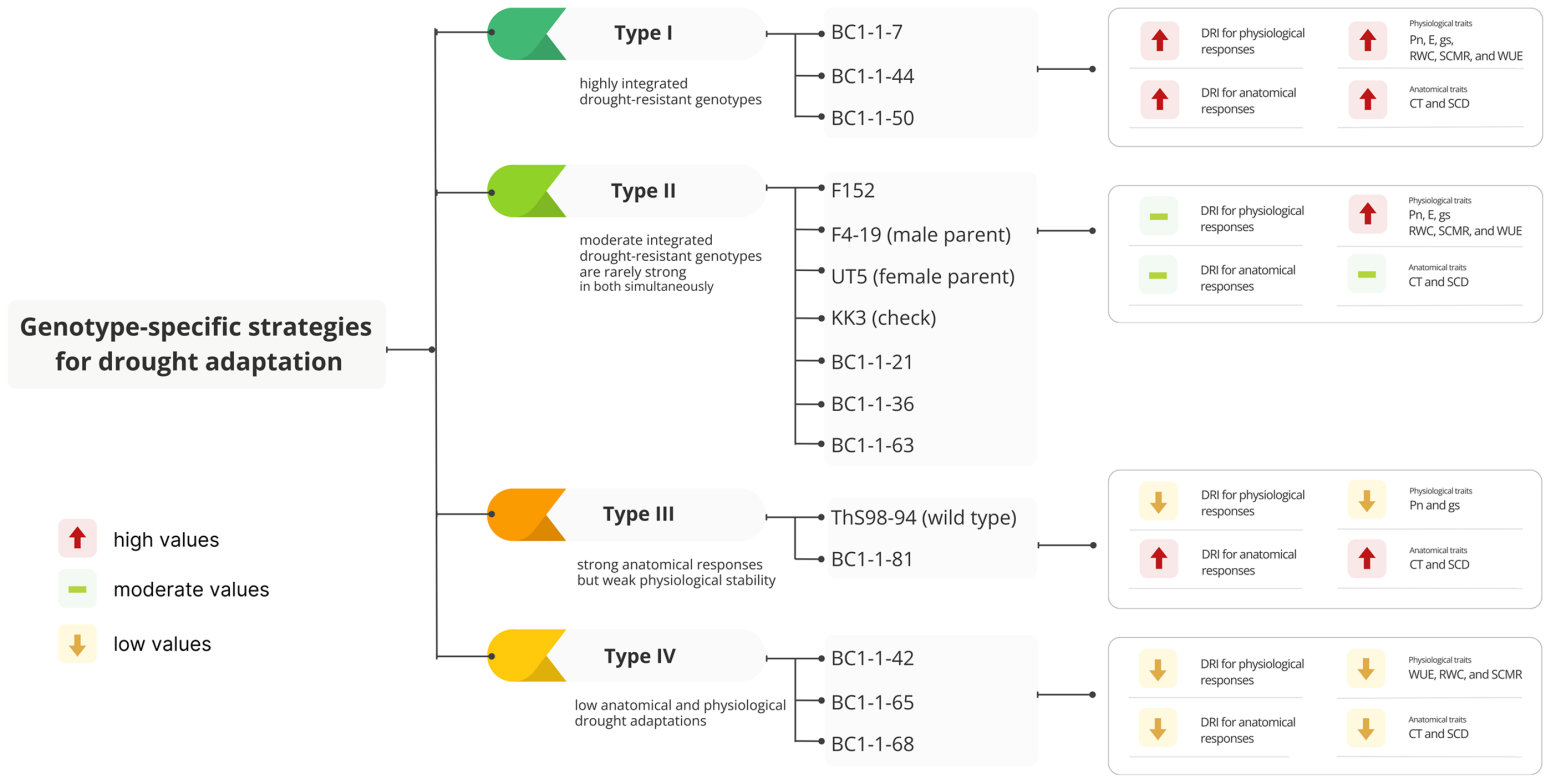
Grouping the genotype-specific strategies into four types based on the integration and strength of their anatomical and physiological traits under drought stress. Genotype-specific strategies for drought adaptation in sugarcane based on the integration and strength of their anatomical and physiological responses: cuticle thickness (CT), stomatal crypt depth (SCD), net photosynthetic rate (*Pn*), transpiration rate (*E*), stomatal conductance to water vapor (*g*s), relative water content (RWC), SPAD chlorophyll meter reading (SCMR), and water use efficiency (WUE) of fifteen sugarcane genotypes under early drought conditions at 110 DAP (days after planting). DRI, drought resistance index.

Based on their anatomical and physiological characteristics, the genotypes in this study showed four distinct drought-response types (Fig. 8). Type I (BC1-1-7, BC1-1-44, and BC1-1-50) exhibited strong integration of anatomical and physiological traits and represented the most drought-resistant candidates for breeding programs. In contrast, Type II (F152, F4-19, UT5, KK3, and several BC1 lines such as BC1-1-21, BC1-1-36, and BC1-1-63) showed moderate drought resistance, with some genotypes displaying strengths in either anatomical or physiological traits but lacking complete integration. Their drought-adaptation strategies appeared genotype-specific, reflecting only partial integration of key traits. For example, F152, UT5, and KK3 showed moderate anatomical changes and stable physiological performance, particularly in SCMR and RWC, and high *P*n and WUE (notably in KK3 and F152). F4-19 demonstrated strong physiological resilience (high *P*n, WUE), traits that were successfully transmitted to progeny such as BC1-1-7 and BC1-1-44. BC1-1-36 and BC1-1-63 showed mixed anatomical and physiological patterns without clear strength in either, though they retained useful traits such as moderate CT and SCD. BC1-1-21 displayed high *P*n and maintained favorable *g*s and *E*, but lacked physiological stability under drought conditions, as indicated by reduced RWC and WUE. Despite these limitations, these Type II genotypes still offer valuable traits for targeted introgression in breeding programs. Type III (ThS98-94 and BC1-1-81) showed strong anatomical adaptation (high SCD, CT) but weak physiological stability, with declines in *P*n and *g*s, and relatively low DRI for physiological traits. ThS98-94 maintained high WUE indicative of wild-type drought-survival traits, while BC1-1-81 showed a similar pattern but would require physiological improvement for breeding use. Type IV (BC1-1-42, BC1-1-65, and BC1-1-68) showed low DRI values for both anatomical and physiological traits, with weak CT and SCD, as well as low WUE, RWC, and SCMR, providing limited drought resistance but potentially contributing to a broader genetic base for improving specific adaptive traits. Among the highly drought-resistant genotypes, BC1-1-7 minimized water loss by combining low LT and VBC with elevated SCD and CT inherited from F4-19 (male parent) and ThS98-94 (wild parent) (Fig. 6), maintaining high WUE while reducing *g*s and *E* (Fig. 7). This water-saving strategy preserved hydration at the cost of some carbon assimilation, making it suitable for environments with sporadic droughts. BC1-1-44, similar to F4-19, sustained high *P*n, *g*s, and *E* under drought, prioritizing photosynthetic output despite increased water loss (Fig. 7). It mildly elevated CT and SCD supported gas exchange and rapid carbon assimilation during brief moisture availability, consistent with its high DRI values for *P*n, *g*s, *E*, and SCMR (Fig. 7). BC1-1-50 employed a mixed strategy, combining high photosynthetic potential inherited from F4-19 with anatomical adjustments such as elevated LT and VBC similar to UT5 (Fig. 6), and maintained a balanced drought response characterized by moderate SCD and WUE (Figs. 6 and 7). BC1-1-7 represents a conservative water-use or isohydric-like strategy, maintaining tighter stomatal control to minimize water loss; BC1-1-44 reflects a more anisohydric-like approach, maintaining stomatal opening longer to maximize carbon assimilation despite declining water potential; and BC1-1-50 shows an intermediate or balanced response between the two extremes. Anisohydric-like traits may have been advantageous for areas with sporadic water availability and balanced traits for versatility genotype, while conservative traits may have produced varieties suitable for extended and severe droughts. By integrating these mechanisms through specific crosses, sugarcane cultivars’ adaptive capacity could be increased, allowing for resilience in diverse drought conditions. Leaf anatomical traits, such as increased cuticle thickness and intact bulliform cells, were indicative of this resilience (Valarmathi et al., 2022). Changes in cuticle thickness under drought stress were crucial for improving WUE and drought tolerance (Valarmathi et al., 2022). Drought-tolerant sugarcane genotypes maintained higher WUE by sustaining elevated net CO_2_ assimilation rates during water scarcity (Contiliani et al., 2023). Therefore, the interplay between cuticle thickness and physiological responses was vital for enhancing drought tolerance and optimizing water use (Misra, Mall & Pathak, 2022; Shrestha, Thapa & Dulal, 2023; Khonghintaisong, Songsri & Jongrungklang, 2023). These anatomical features enabled hybrid sugarcane plants to withstand water deficit stress, ensuring survival and productivity in challenging environments. Drought stress reduced leaf thickness, affecting stomatal conductance (Khan et al., 2023). BC1-1-7 and BC1-1-50 showed greater overall drought resistance in physiological parameters, such as low stomatal conductance and transpiration rate, but exhibited high WUE compared to BC1-1-44, indicating an effective water management strategy under limited water availability. Variability in stomatal conductance was influenced by factors such as leaf mass per area (LMA) and leaf water content (Zou et al., 2022). Species-specific responses to aridity included changes in leaf thickness, LMA, and transpiration rate (Ronzhina, Ivanova & Ivanov, 2019). Germplasm from interspecific hybrids could have significantly enhanced sugar production by introducing genetic diversity, improving specific traits, and ensuring adaptability to various environments (Ngaklunchon et al., 2023).

Overall, these mechanisms contributed to the increased drought resistance of BC1 interspecific hybrids. These traits indicated their ability to cope with water scarcity and maintain photosynthetic activity under drought stress. Improved photosynthetic efficiency often translated to higher biomass production, with some hybrids showing high photosynthesis rates in both early and late seasons, correlating with increased biomass yield (Kar et al., 2020). Further studies and field trials were recommended to validate these findings and assess the performance of these genotypes under actual drought conditions.

## Conclusions

This is the first report investigating the anatomical responses of BC1 interspecific hybrids generated by crossing commercial cane with wild-type sugarcane under drought conditions. The genotypes BC1-1-7, BC1-1-44, and BC1-1-50 demonstrated superior drought resistance and adaptive traits, exhibiting higher DRI for both tiller height and number. Anatomical traits in BC1-1-7 and BC1-1-44 resemble their male parent (interspecific F1 hybrid) in terms of LT, CT, and VBC, while BC1-1-50 shows adaptations similar to its female parent (commercial cane). These adjustments help conserve water and reduce transpiration, enabling the plants to withstand drought conditions. Moreover, BC1-1-7 and BC1-1-50 exhibited low *g*s and *E*, as well as high WUE compared to BC1-1-44 but less than the F4-19 and KK3, indicating that they continue to use water efficiently even when there is a shortage. Overall, BC1-1-7 demonstrated the most favorable physiological and anatomical characteristics for drought resistance relative to BC1-1-44 and BC1-1-50. The findings would contribute to the further development of sugarcane varieties while incorporating commercially desirable traits.

##  Supplemental Information

10.7717/peerj.20822/supp-1 Supplemental Information 1 Raw data of weather, soil moisture content, physiology, morphology and anatomy

## Additional Information and Declarations

### Data Availability

The following information was supplied regarding data availability: The raw measurements are available in the Supplemental File.
